# Educational Attainment and Social Engagement in Later Life: A Cohort Analysis

**DOI:** 10.1093/geroni/igaf122.111

**Published:** 2025-12-31

**Authors:** James Iveniuk, Alyssa Goldman, Markus Schafer

**Affiliations:** National Opinion Research Center at The University of Chicago, Scarborough, Ontario, Canada; Boston College, Chestnut Hill, Massachusetts, United States; Baylor University, Waco, Texas, United States

## Abstract

Associations between educational attainment and social capital are well established, however, this relationship has not been explored in the context of the changing educational landscape in the United States. Since before the 1950s, rates of college attendance and college degree completion have dramatically increased, especially among women. These trends raise questions about whether the advantages of higher education for social capital have remained consistent or exhibit heterogeneity across birth cohorts and forms of social engagement. We use four rounds of U.S. population-based data from the National Social Life, Health, and Aging Project in a series of multilevel models to estimate the effects of older adults’ educational attainment on six different measures of social engagement. We pay particular attention to differences across birth cohorts between 1930 and 1965. Our findings indicate that higher levels of education are associated with significantly more frequent volunteering, socializing, religious service attendance, and participating in organized groups, as well as having more friends and larger discussion networks in later life. These findings emerge when adjusting for parental education, age, gender, and race/ethnicity. Associations between education and network size, number of friends, and number of close relatives, are stronger among earlier-born compared to later-born cohorts. However, education is more weakly associated with the frequency of socializing for earlier-born cohorts. We discuss potential mechanisms that underlie these associations, including the alignment of the cohort differences with other social and institutional phenomena in the U.S. We consider the implications for efforts to address social isolation and socioeconomic health disparities.

